# TGM3, a candidate tumor suppressor gene, contributes to human head and neck cancer

**DOI:** 10.1186/1476-4598-12-151

**Published:** 2013-12-01

**Authors:** Xiangbing Wu, Wei Cao, Xu Wang, Jianjun Zhang, Zhongjing Lv, Xing Qin, Yadi Wu, Wantao Chen

**Affiliations:** 1 Department of Oral and Maxillofacial-Head and Neck Oncology and Facuty of Oral and Maxillofacial Surgery, Ninth People’s Hospital, Shanghai Jiao Tong University School of Medicine, Shanghai 200011, China; 2 Shanghai Research Institute of Stomatology and Shanghai Key Laboratory of Stomatology, Shanghai 200011, China

**Keywords:** Transglutaminase 3, Head and neck cancer, Tumor suppressor, Methylation, Prognostic predictor

## Abstract

**Background:**

In our previous study using oligonucleotide microarrays, we revealed that transglutaminase 3 (TGM3) was remarkably down-regulated in head and neck cancer (HNC). However, the potential of TGM3 as a useful biomarker or molecular target for HNC is unclear.

**Methods:**

The transcriptional and post-translational status of TGM3 in HNC cell lines and specimens was detected using real-time PCR and western blot analysis. Bisulfate-treated DNA sequencing was used to analyze the molecular mechanism of TGM3 gene silencing. In addition, the effects of TGM3 on the proliferation, colony formation and induction of apoptosis *in vitro* and tumorigenicity *in vivo* were investigated through exogenous expression of TGM3 in HNC cells. Immunohistochemistry was used to evaluate TGM3 expression in large HNC samples.

**Results:**

TGM3 was down-regulated in HNC samples and cell lines (*P* < 0.0001). The hypermethylation of a promoter CpG island was one of the mechanisms of silencing the TGM3 gene in HNC. Exogenous expression of TGM3 in HNC cells could inhibit the proliferation and enhance the apoptosis of HNC cells *in vitro* and suppress tumor growth *in vivo*. In addition, TGM3 protein levels were strongly associated with the pathological differentiation of HNC tissues (*P* = 0.0037). Survival analysis revealed that low TGM3 expression was associated with worse overall survival (*P* = 0.0002), and TGM3 expression level was an independent predictor in patients with HNC.

**Conclusions:**

The studies prove that TGM3, as a candidate tumor suppressor, contributes to the carcinogenesis and development of HNC and may serve as a useful biomarker for patients with HNC.

## Introduction

Head and neck cancer (HNC), squamous cell carcinoma accounts for over 90%, is the sixth most common cancer in the world [1]. Despite continuous improvement in traditional treatments (surgery, radiotherapy and chemotherapy), the 5-year survival rate for patients with these devastating diseases has been unsatisfactory in the past three decades [2]. In an attempt to improve the outcomes of HNC, the application of novel and effective biomarkers for diagnosing and predicting HNCs is so important and urgent. Recently, certain molecular biomarkers have been found to have promising diagnostic and predicting potentials and are already used in oncological practice [3-6], whereas others necessitate further studies. As we have known, the development of HNC is a multistep carcinogenic processes that includes activation of several oncogenes and inactivation of tumor suppressor genes [7]. High-throughput microarray technology might be an efficient way to uncover clues to these processes and find biomarkers for the diagnosis, therapy and prognosis of HNC [8-10]. In our previous study [11], oligonucleotide microarrays (Affymetrix HG-U95Av2) were used to select differentially expressed genes between 22 pairs of head and neck squamous cell carcinoma (HNSCC) and normal epithelial tissues from the same donors. Remarkably, transglutaminase 3 (TGM3) was significantly down-regulated in HNSCCs.

TGM3, encoded by the TGM3 gene, is widely expressed in the small intestine, brain, skin and mucosa [12]. In the skin and mucosa, TGM3 is predominantly expressed in the suprabasal layers of the stratified squamous epithelium [13,14]. It has been demonstrated that TGM3 is essential for epidermal terminal differentiation and formation of the cornified cell envelope through cross-linking structural proteins such as involucrin, loricrin and small proline-rich proteins [15,16]. Recent studies have revealed that the down-regulation of the TGM3 gene is closely linked with a variety of human cancer types, including laryngeal carcinoma, esophageal and oral squamous cell carcinoma (OSCC) [17-19]. Moreover, Uemura et al. reported that TGM3 was identified as a novel prognostic indicator in ESCC and the prognostic performance of TGM3 was confirmed by immunohistochemistry in 76 ESCC cases [18]. In addition, Mendez et al. reported that the TGM3 gene was differentially expressed in node-positive and node-negative primary tumors in patients with OSCC, implying that the decreasing TGM3 expression might contribute to the metastatic potential of OSCC [20]. However, the biological function and molecular mechanism of the TGM3 gene in cancer initiation and progression have not been reported. In addition, whether the TGM3 gene might be a valuable diagnostic or therapeutic biomarker for cancer, especially for HNC, needs to be further investigated.

In our current study, we confirmed that the transcriptional and post-translational levels of TGM3 were down-regulated in HNSCC cell lines and specimens compared with normal primary head and neck epithelial cells and paired adjacent normal tissues, by means of the real-time RT-PCR, semi-quantitative RT-PCR and western blotting. We further found that the hypermethylation of a promoter CpG island was one of the mechanisms contributing to the silencing of the TGM3 gene in HNSCC. Furthermore, we evaluated the effect of ectopic TGM3 expression on WSU-HN4, HN13 and HN30 cells, which are HNSCC-derived cell lines that lack endogenous TGM3 expression. We first provide evidence that the exogenous expression of TGM3 in HNSCC cell lines inhibits the proliferation, colony formation and induces the apoptosis in cancer cells *in vitro* and tumorigenicity *in vivo*. In addition, we demonstrate that low TGM3 expression is notably associated with poorly differentiated tumors and worse overall survival. These results suggest that TGM3 might be a candidate tumor suppressor contributing to HNC and could act as a valuable prognostic predictor for patients with HNC.

## Results

### TGM3 is down-regulated in HNSCC samples and cell lines

**Figure 1:**
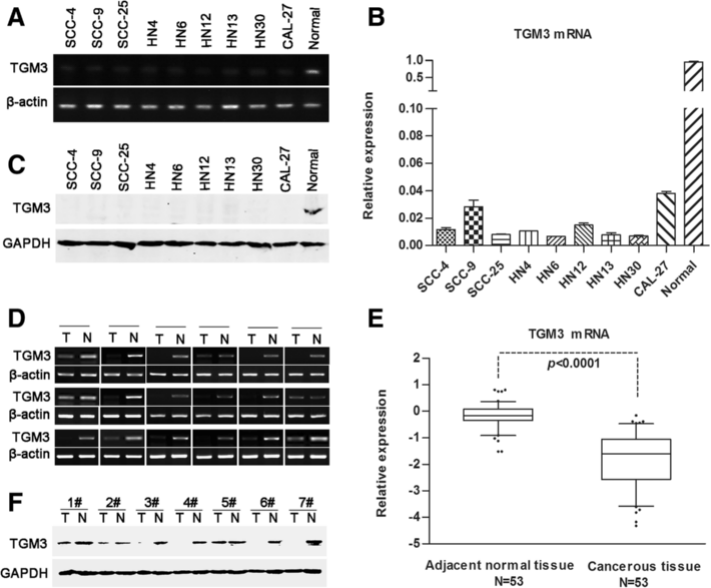
**TGM3 mRNA and protein levels are illustrated in HNSCC specimens and cell lines. (A, B, C)** TGM3 transcriptional and translational status in 9 representative HNSCC cell lines and primary normal epithelial cells (titled *normal*) was determined using semi-quantitative RT-PCR, real-time PCR and western blotting analysis. **(D)** Representative results of TGM3 mRNA levels from 18 pairs of HNSCC and their adjacent normal tissue. **(E, F)** TGM3 mRNA and protein levels were determined in 53 paired samples from patients with HNSCC and in 7 paired HNSCC samples using real-time PCR and western blot analyses (*N*, adjacent normal tissue; *T*, tumor tissue).

To confirm our high-throughput microarray data, semi-quantitative RT-PCR and real-time RT-PCR analyses were performed to investigate the mRNA levels of TGM3 in 53 paired HNSCC specimens, 9 HNSCC cell lines, and normal primary head and neck epithelial cells. The TGM3 mRNA levels in the HNSCC cell lines were significantly lower than those of normal epithelial cells (Figure 1A and B). Meanwhile, TGM3 transcription levels were remarkably down-regulated in HNSCC tissues compared with the levels of paired adjacent normal tissues (*P* < 0.0001) (Figure 1D and E). Correspondingly, the protein levels of TGM3 were remarkably down-regulated in 9 HNSCC cell lines and 7 HNSCC tissues compared with the levels in normal epithelial cells and the paired adjacent normal tissues using western blot analysis (Figure 1C and F).

### The transcription of TGM3 is regulated by DNA promoter methylation in HNSCC samples and cell lines

**Figure 2:**
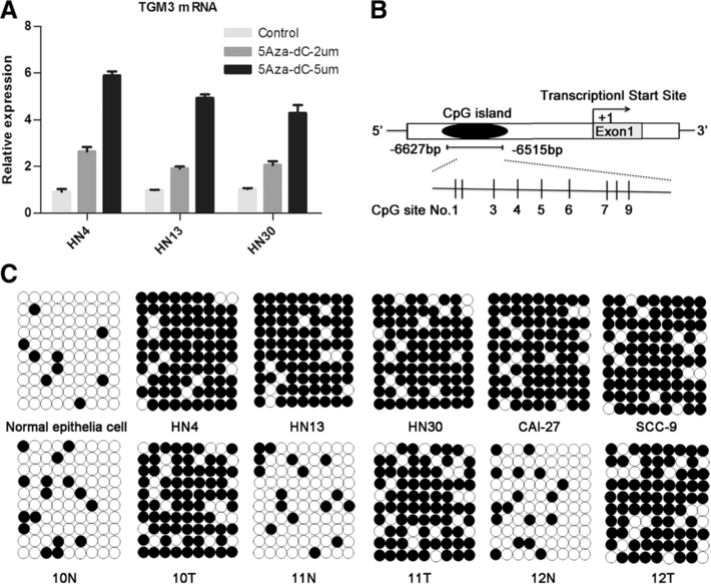
**Promoter region hypermethylation mediated the silencing of TGM3 in HNSCC. (A)** Restoration of TGM3 expression by 5-Aza-dC. Three representative HNSCC cell lines were treated with 2 μM or 5 μM 5-Aza-dC for 5 days, and TGM3 transcriptional levels were determined by real-time RT-PCR. **(B)** Schematic representations of the location of the CpG islands in the upstream region of the TGM3 gene and the location of bisulfate-based sequencing within the CpG island. **(C)** Bisulfate-treated genomic DNA sequencing to detect the methylation status of the CpG islands in 5 HNSCC cell lines and 3 representative HNSCC samples compared with the methylation status in normal primary head and neck epithelial cells and paired adjacent normal tissues. White and black circles represent unmethylated and methylated CpGs, respectively.

To investigate the molecular mechanism of TGM3 gene silencing in HNSCC tumor samples and cell lines, all of the above-mentioned HNSCC cell lines lacking TGM3 expression were treated with the demethylation agent 5-Aza-dC and the histone deacetylase inhibitor TSA. Real-time PCR analysis revealed a dose-dependent up-regulation of TGM3 mRNA expression after treatment with 5-Aza-dC (Figure 2A). However, there was no effect on the reactivation of TGM3 expression after treatment with TSA, indicating that histone deacetylation does not contribute to the transcriptional silencing of TGM3 in HNSCC cell lines (data not shown). To further confirm that the TGM3 gene was regulated through DNA hypermethylation, a putative CpG island, consisting of a 113 bp fragment containing 9 CpG sites located −6627 to −6515 bp relative to the transcriptional start site (TSS) was found by the MethPrimer online software (Figure 2B). Next, bisulfate-treated DNA sequencing was performed to examine the DNA methylation status of this CpG islands in 5 randomly selected HNSCC cell lines and 5 pairs of HNSCC tissues from 53 cases. The results showed that this CpG island was hypermethylated in the HNSCC cell lines relative to normal primary head and neck epithelial cells. Moreover, the methylation level of the CpG island was significantly higher in all 5 HNSCC samples than in the paired adjacent normal tissues (*P* < 0.05, Figure 2C).

### Correlation between TGM3 protein level and clinicopathologic parameters and clinical outcome in patients with HNSCCs

**Figure 3:**
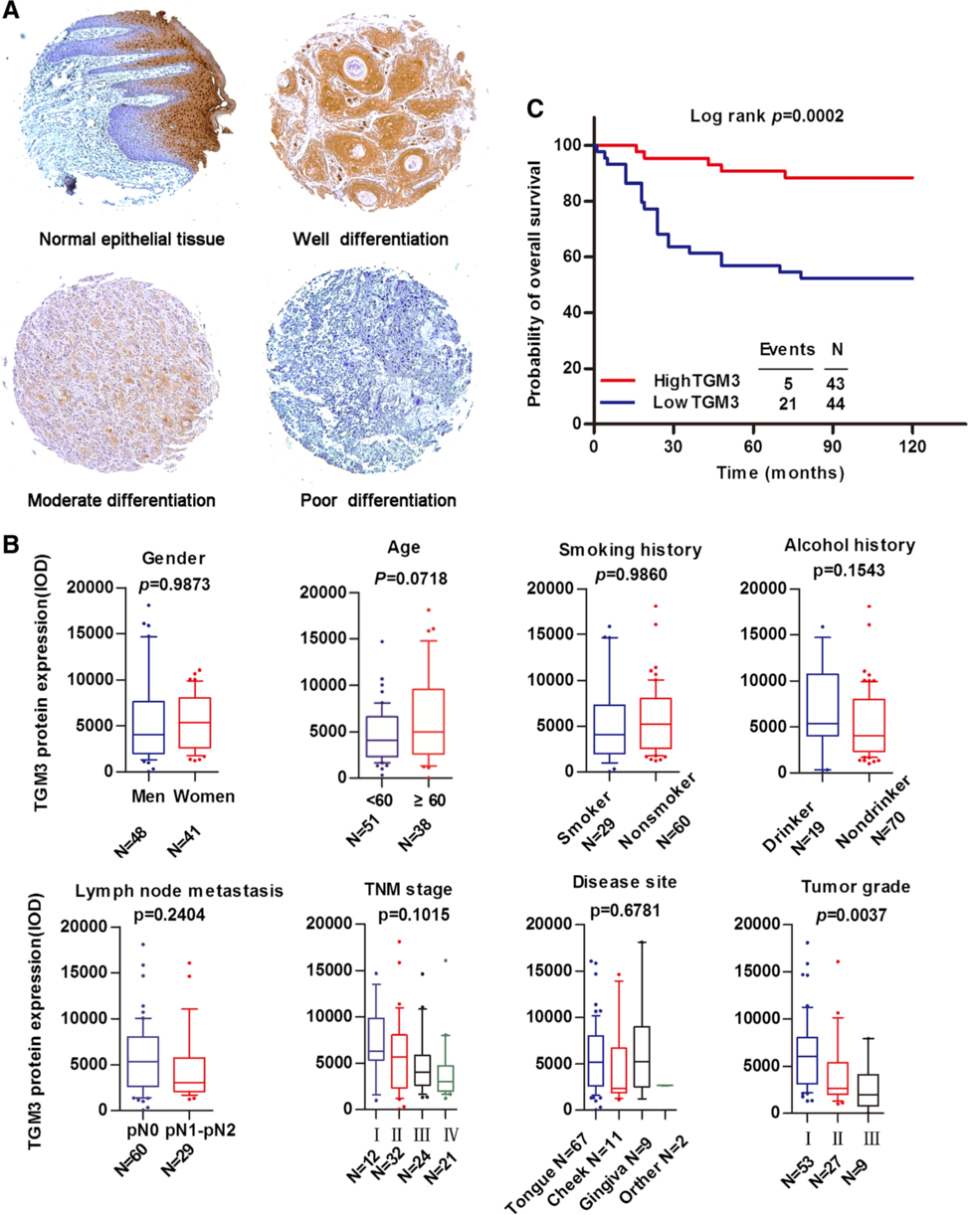
**Correlation between TGM3 expression and clinicopathological parameters and clinical outcome in patients with HNSCCs. (A)** Representative images show immunohistochemical staining for TGM3 expression in normal epithelial tissue, well differentiated HNSCC, moderately differentiated HNSCC and poorly differentiated HNSCC. **(B)** The associations between TGM3 expression and clinicopathological factors were investigated in patients with HNSCCs (Twelve patients were excluded due to the samples shedding in the process of antigen retrieval and due to an insufficient number of tumor cells in the sections). **(C)** Kaplan-Meier survival curves illustrate the overall survival of patients with HNSCC relative to TGM3 expression (Two patients were excluded from survival analysis because of missing follow-up data).

Using immunohistochemical staining to detect TGM3 expression levels, we observed that, in normal epithelial tissue, cytoplasmic and nuclear staining of TGM3 was expressed in keratinized layer, stratum granulosum and in a portion of stratum spinosum nearby stratum granulosum. Whereas weak or no staining of TGM3 was observed in basal layer and in a portion of stratum spinosum nearby basal layer. In HNSCC specimens, cytoplasmic and nuclear staining of TGM3 was detected in well differentiated tumors, whereas weak or no staining of TGM3 was observed in moderately differentiated and poorly differentiated tumors (Figure 3A). There was positive association between TGM3 expression level and the pathological differentiation of the tumors (*P* = 0.0037), whereas no significant associations were determined between TGM3 expression pattern and gender (*P* = 0.9873), age (*P* = 0.0718), alcohol history (*P* = 0.1543), smoking history (*P* = 0.9860), TNM classification (*P* = 0.1015), lymph node metastasis (*P* = 0.2404), or anatomic site (*P* = 0.6781, Figure 3B). Overall survival probability was estimated using Kaplan-Meier survival analysis to determine the correlation between TGM3 expression and clinical outcome in patients with HNSCCs. We observed that patients whose primary tumors expressed a low level of TGM3 had significantly poorer overall survival (*P* = 0.0002, Figure 3C). In univariate and multivariate Cox proportional analyses (Table 1), TGM3 expression status (hazard ratio, 0.286; 95% CI, 0.103-0.796; *P* = 0.016), together with pathological differentiation of the tumor (hazard ratio, 1.737; 95% CI, 1.042-2.895; *P* = 0.034), was identified as an independent predictor of clinical outcome in patients with HNSCCs.

**Table 1:** Univariate and multivariate cox proportional hazards regression models for estimating overall survival

**Characteristics**	**HR**	**95% CI**	***P***
**Univariate analysis**			
Overall survival			
Age (<60 y vs ≥ 60 y)	0.633	0.282 to 1.419	0.267
Gender (male vs female)	1.713	0.763 to 3.844	0.192
Smoking history (smoker vs nonsmoker)	0.643	0.295 to 1.401	0.267
Alcohol history (drinker vs nondrinker)	0.718	0.302 to 1.709	0.454
Tumor grade	2.096	1.283 to 3.425	0.003
TNM stage	1.784	1.181 to 2.696	0.006
Lymph node metastasis (pN0 vs pN1 to pN2)	2.514	1.153 to 5.481	0.020
TGM3 expression (high vs low)	0.191	0.072 to 0.506	0.001
Disease site	1.023	0.637 to 1.660	0.926
**Multivariate analysis**			
Overall survival			
Tumor grade	1.737	1.042 to 2.895	0.034
TNM stage	1.325	0.806 to 2.177	0.267
Lymph node metastasis (pN0 vs pN1 to pN2)	1.515	0.586 to 3.909	0.391
TGM3 expression (high vs low)	0.286	0.103 to 0.796	0.016

*Abbreviations*: *CI* confidence interval; *HR* hazard ratio; *pN* pathological lymph node status; *TNM* tumor-lymph node-metastasis classification.

### TGM3 Inhibits cell proliferation and colony formation

**Figure 4:**
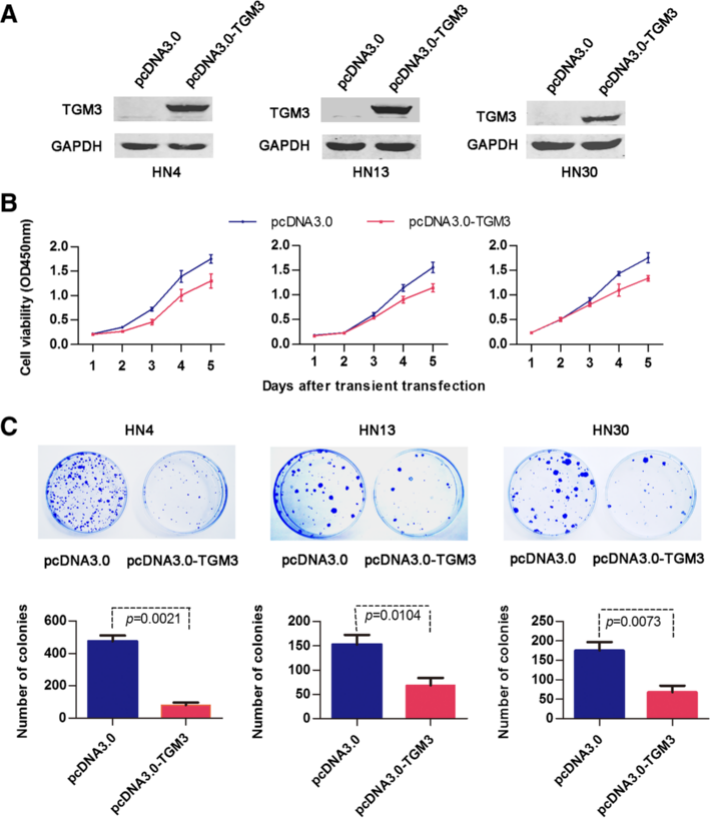
**The effect of TGM3 on cell growth and colony formation of HNSCC cell lines. (A)** Western blot analysis confirmed the exogenous expression of TGM3 in HN4, HN13 and HN30 cells. **(B)** The growth of HN4, HN13 and HN30 cells transfected with the pcDNA3.0 and pcDNA3.0-TGM3 vectors were analyzed using the CCK-8 kit; the symbols represent the mean values of triplicate tests (mean ± SD). **(C)** Representative dishes show the inhibitory effect of TGM3 on colony formation compared with the empty vector control. All experiments were repeated at least 3 times.

To analyze the function of the TGM3 protein in HNSCC cells, we transiently transfected mammalian expression vectors containing TGM3 into the WSU-HN4, HN13 and HN30 cell lines, all of which lack endogenous TGM3 expression (Figure 4A). Our results showed that exogenous TGM3 expression notably suppressed the proliferation and colony-formation abilities of these cells relative to mock-transfected controls (Figure 4B and C). To further evaluate whether cell cycle distribution was altered, flow cytometry was performed in cells expressing exogenous TGM3. Nevertheless, there was no difference in cell cycle distribution between the cells transiently transfected with the pcDNA3.0-TGM3 vector and the pcDNA3.0 empty vector (data not shown).

### Induction of apoptosis in HNSCC cells by ectopic TGM3 expression

**Figure 5:**
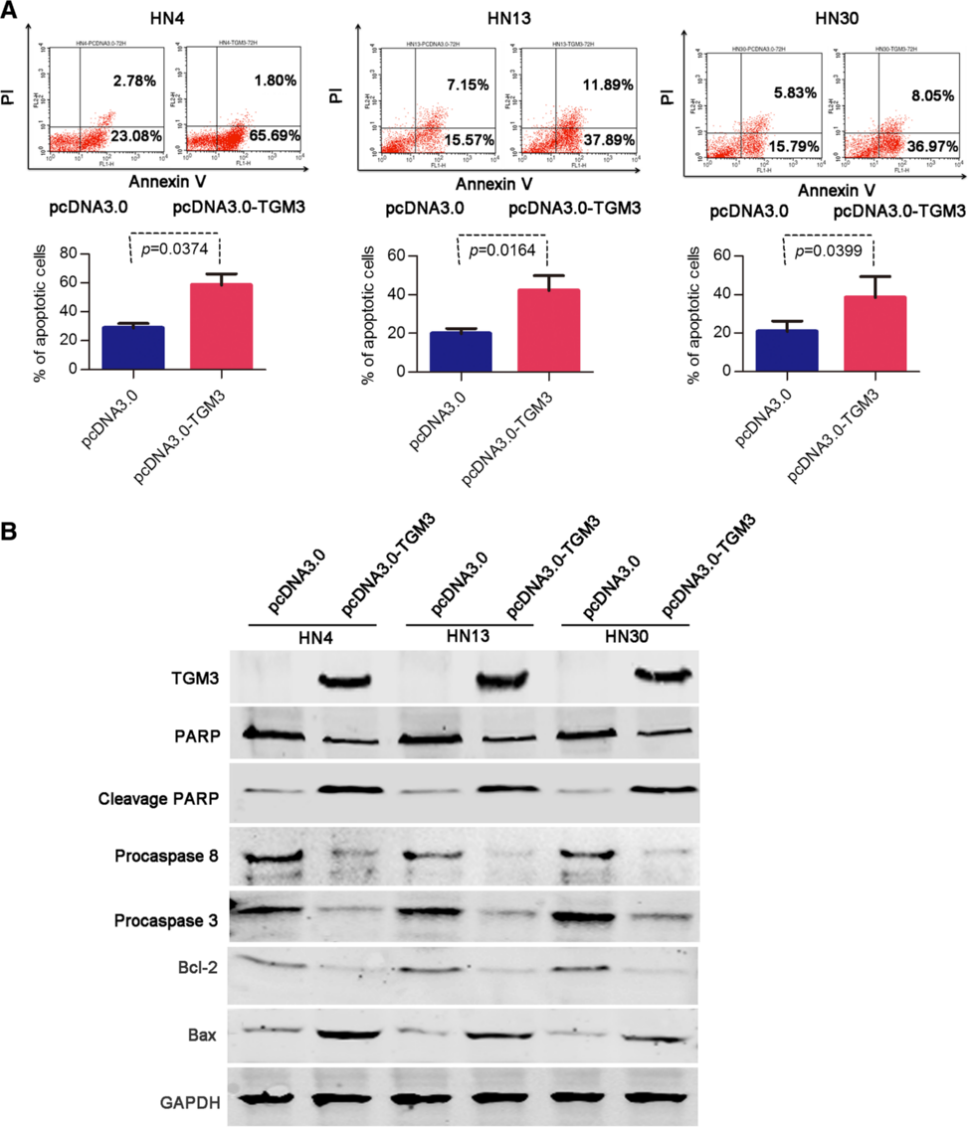
**The effect of TGM3 on HNSCC cell apoptosis. (A)** The proportion of apoptotic cells was markedly increased (lower right fraction) in TGM3-transfected HN4, HN13 and HN30 cells, relative to the mock-transfected control cells, as detected by PI and Annexin V staining. **(B)** The change in the apoptosis-related proteins full-length PARP, cleaved PARP, procaspase-3, procaspase-8, Bcl-2 and Bax was assessed by western blotting after transient transfection with the pcDNA3.0-TGM3 and pcDNA3.0 vector for 72 hours.

To analyze whether the suppressive effect of the TGM3 protein on cells was caused by apoptosis, the fraction of TGM3-transfected WSU-HN4, HN13 and HN30 cells with positive staining for propidium iodide (PI) and Annexin V were assessed by flow cytometry. The fractions of apoptotic cells (lower right (LR) fraction) in TGM3-transfected WSU-HN4, HN13 and HN30 cells were 65.69%, 37.89% and 36.97%, respectively, which were notably higher than those of the mock-transfected control cells (23.08%, 15.57%, 15.79%) (*P* < 0.05, Figure 5A). Furthermore, we also analyzed differences in specific apoptosis-related proteins between cells transiently transfected with the pcDNA3.0-TGM3 vector and the pcDNA3.0 empty vector. As shown in Figure 5B, the protein levels of cleaved PARP and Bax were increased and the levels of full-length PARP, procaspase-3, procaspase-8 and Bcl-2 were reduced in TGM3-transfected HN4, HN13 and HN30 cells, compared to mock-transfected control cells, as detected by western blotting analysis.

### Ectopic expression of TGM3 suppresses tumor growth *in vivo*

**Figure 6:**
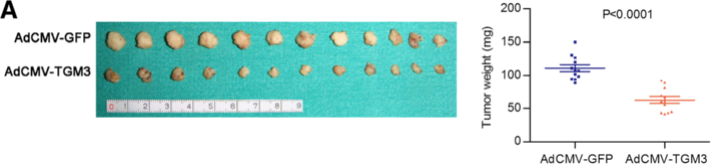
**The effect of TGM3 on tumorigenicity *in vivo*. (A)** Exogenous TGM3 expression inhibited xenograft tumor growth of HN30 cells treated with AdCMV-TGM3 relative to HN30 cells treated with AdCMV-GFP. Tumors’ weights were measured (mean ± SD).

After evaluating the anti-tumor activity of TGM3 *in vitro*, we next analyzed whether exogenous TGM3 expression affected tumorigenicity *in vivo* using an HN30 xenograft model in BALB/C nude mice. One week after subcutaneously injecting HN30 cells, AdCMV-TGM3 and AdCMV-GFP was administered locally by multiple-center intratumor injection. As shown in Figure 6, the average tumor sizes with AdCMV-TGM3 treatment were significantly smaller than the control group (*P* < 0.0001).

## Discussion

The limitations of conventional treatments in advanced, recurrent or distant metastases HNC have necessitated a search for novel potential approaches. Recently, several research groups have reported certain genes and signaling molecules that were potentially involved in HNC initiation and progression [21-24]. These molecular targets could provide significant clues for early diagnosis, prognosis and new targeted therapies [25-30]. In our previous study [11], we found that TGM3 was remarkably down-regulated in HNSCC compared with adjacent normal tissues by means of an oligonucleotide microarray analysis. In this study, we have first verified the microarray data by detecting the mRNA and protein levels of TGM3 in 9 HNSCC cell lines and 53 HNSCC specimens. Our findings were consistent with other reports, in which TGM3 was also found to be down-regulation in laryngeal carcinoma, esophageal SCC and OSCC [31-40]. Furthermore, we evaluated the clinical significance of TGM3 by analyzing the correlation between TGM3 expression pattern and clinicopathologic parameters and clinical outcome in patients with HNSCC. Our data demonstrated that the reduced expression of TGM3 in HNSCC was associated with poorer pathological tumor differentiation (*P* = 0.0037), consistent with the findings described in ESCC and OSCC [19,38]. Moreover, we observed that patients whose tumors expressed a low level of TGM3 had worse overall survival (*P* = 0.0002) and that TGM3 expression, by univariate and multivariate analyses, was an independent prognostic factor in patients with HNSCC. This result was consistent with the findings of Uemura, who reported that the 5-year disease-specific survival rate was 64.5% and 32.1%, respectively, for patients with TGM3-positive and TGM3-negative ESCC, and TGM3 was identified as independent predictor in patients with ESCC [18]. Although Mendez et al. reported that TGM3 expression was inversely correlated with lymph node metastasis of OSCCs [20], we did not observe a significant correlation between TGM3 expression and lymph node metastasis in our study. This inconformity is most likely due to the different type and number of tumor samples included in the different studies. In Mendez’s research, the number of OSCC specimens was 11 and the type of samples selectively included 6 metastatic lymph node samples. In our study, the tumor specimens were 101 cases which were all primary HNSCC samples and the exclusion criteria of these samples included preoperative radiotherapy and/or chemotherapy, distant metastasis and recurrent tumors.

Although TGM3 down-regulation has been found in many cases, the molecular mechanism that causes the silencing of TGM3 expression in most cancers remains unclear. He et al. reported that the loss of heterozygosity within and near the TGM3 gene might lead to down-regulation of TGM3 in laryngeal carcinoma [17]. Aside from genetic changes, epigenetic alterations including DNA methylation, histone modification and non-coding RNA regulation could account for the down-regulation of TGM3. To evaluate the regulation of the DNA methylation and histone deacetylation modifications on TGM3 expression in HNSCC, we treated HNSCC cell lines with the demethylation agent 5-Aza-dC and the histone deacetylase inhibitor TSA. Our results, consistent with Negishi’s findings [19], showed a dose-dependent up-regulation of TGM3 mRNA expression after treatment with 5-Aza-dC and no change in TGM3 expression after treatment with TSA. Although TGM3 gene hypermethylation in OSCC cell lines was first found by Negishi et al. [19], a more detailed characterization of the DNA hypermethylation status of the TGM3 gene promoter in paired HNSCC samples and adjacent normal tissues has never been reported. Bioinformatics tools were utilized to search for putative CpG islands in the regulatory region of the TGM3 gene from 8,000 bp upstream to 500 bp downstream of the transcriptional start site. We have first identified a CpG island located at −6627 to −6515 bp (including 9 CpG sites) relative to the transcriptional start site. Furthermore, bisulfate-treated DNA sequencing revealed that the CpG island was hypermethylated in 5 HNSCC cell lines relative to normal primary head and neck epithelial cells, and the methylation level of this CpG island was significantly higher in 5 randomly selected HNSCC samples than in paired adjacent normal tissues. Based on our results and the findings reported by others [19], we propose that DNA hypermethylation in the TGM3 regulatory region contributes, at least partially, to the down-regulation of TGM3 in HNSCC.

Transglutaminases (TGMs) are a family of calcium-dependent enzymes that catalyze the formation of isopeptide bonds [41,42]. TGM3 is epidermal transglutaminase, which is expressed predominantly in the suprabasal layers of the stratified squamous epithelium [43]. Although, the biological function of TGM3 in stratified squamous epithelium is very clear, *in vitro* and *in vivo* functional experiments of TGM3 on epithelial cancer cells have never been reported. In this study, we evaluated the functional role of the TGM3 gene in 3 HNSCC cell lines. Our results, for the first time, demonstrated that exogenous TGM3 expression could exert strong anti-proliferative effects on HNSCC cell lines *in vitro* and decreased tumorigenicity *in vivo*. Although, no difference in cell cycle distribution was detected between the cells transiently expressed TGM3 protein or not. This unanticipated result is most likely due to that exogenous TGM3 expression in HNSCC cell lines does not affect the cell cycle-related proteins such as cyclin D1, p53 and CDK1. We propose that the suppressive effect on tumorigenicity is possibly a result of the remarkable induction of cell apoptosis. The reduced protein level of full-length PARP, procaspase-3, procaspase-8 and Bcl-2 and the increased protein level of cleaved PARP and Bax in the TGM3-expressing HNSCC cell lines, as shown by western blot analysis, confirmed that the induction of apoptosis is closely involved in the effects of ectopic TGM3 expression. However, further study is required to elucidate its precise mechanism.

## Conclusions

In summary, our study has shown that the silencing of TGM3 by DNA hypermethylation is functionally involved in HNSCC cell proliferation and apoptosis. Moreover, in this study, we have shown that TGM3 expression is significantly associated with pathological differentiation of HNSCC and that TGM3 expression is an independent predictor of clinical outcome in patients with HNSCC. Our results strongly suggest that the TGM3 may be a candidate tumor suppressor and may act as a potential and novel biomarker for HNC.

## Materials and methods

### Cell culture

As previously described [44], the human head and neck squamous cell carcinoma cell lines WSU-HN4, HN6, HN12, HN13 and HN30 (kindly provided by the University of Maryland Dental School, USA) and CAL-27 (American Type Culture Collection, ATCC, USA) were cultured in Dulbecco’s modified Eagle medium (DMEM; GIBCO-BRL, USA) supplemented with 10% heat-inactivated FBS (GIBCO-BRL), penicillin (100 units/ml), and streptomycin (100 μg/ml) at 37°C in a humidified 5% CO2 atmosphere. SCC-4, SCC-9 and SCC-25 cells (purchased from the ATCC) were cultured in DMEM/F12 medium (GIBCO-BRL) supplemented with 10% heat-inactivated FBS, penicillin (100 units/ml), and streptomycin (100 μg/ml). Normal primary head and neck epithelial cells were cultured in keratinocyte serum-free medium (KSF; GIBCO-BRL) with 0.2 ng/mL recombinant epidermal growth factor (rEGF; Invitrogen, USA).

### Patients and specimens

Samples were obtained from a cohort of 53 patients who were diagnosed with primary HNSCC and underwent initial surgical treatment in the Department of Oral and Maxillofacial-Head and Neck Oncology, Ninth People’s Hospital, Shanghai Jiao Tong University School of Medicine (Shanghai, China) between June 2008 and December 2009. Half of each sample was embedded in paraffin for pathologic examination and immunohistochemical staining, and the other half was quickly frozen in liquid nitrogen until total RNA and protein was extracted. In addition, a tissue microarray was constructed with a donor block of paraffin-embedded tissues from 101 patients who were histologically diagnosed with primary HNSCC and underwent initial surgical treatment between 1989 and 1993. The medical records of the patients were retrospectively reviewed for inclusion and exclusion criteria. Exclusion criteria included preoperative radiotherapy and/or chemotherapy, distant metastasis, recurrent tumors and incomplete medical records. The clinicopathologic characteristics of 101 patients are summarized in Additional file 1: Table S1. All 101 patients had clinical information and 99 patients had follow-up data. The median follow-up time was 73 months. In this study, samples from each patient were stained with hematoxylin and eosin, classified histologically, and staged according to the TNM classification system of the International Union Against Cancer (1988) prior to further analysis. The study was approved by the Institutional Ethics Committee of the Ninth People’s Hospital, Shanghai Jiao Tong University School of Medicine (number: 2012–91) and all of the patients signed written informed consent in accordance with the institutional guidelines.

### Immunohistochemical analysis

Immunohistochemical staining was performed using a rabbit polyclonal TGM3 antibody (Sigma, USA 1:50). The avidin-biotin complex (ABC) technique was used following the manufacturer’s instructions for the Vectastatin elite ABC kit (Vector Laboratories, USA). Briefly, tissue microarrays were deparaffinized in xylene, rehydrated in graded ethanol, treated with citrate buffer for heat-induced epitope retrieval, incubated overnight at 4°C with rabbit polyclonal TGM3 antibody followed by incubation with a biotinylated secondary antibody and ABC reagent. Twelve patients were excluded due to the samples shedding in the process of antigen retrieval and an insufficient number of tumor cells in the sections. Image-pro plus 6.0 (IPP 6.0) software was used to quantitatively determine the integrated optical density (IOD) of TGM3 staining. The procedure was performed as described in our previous study [45]. TGM3 expression was determined by randomly selecting five tumor cell areas of each specimen under the same conditions including light source, gain, brightness, color saturation, and contrast, then the function of irregular automated optical inspection was used by IPP 6.0 software to score. The median score of all specimen was 4683 (range 51–18083), which was set as the cut-off value to determine high or low TGM3 expression; thus, a value >4683 was considered high expression and a value ≤ 4683 was considered low expression.

### Semiquantitative reverse transcriptase-polymerase chain reaction analysis

Total RNA was extracted with Trizol reagent (Invitrogen) and 1 μg total RNA was reverse transcribed into complementary DNA (cDNA) in a total volume of 20 μl using the PrimerScript RT reagent Kit (Takara, China). One microliter of each RT reaction mixture was then subjected to PCR amplification using EX Taq DNA polymerase (Takara). The primer sequences were as follows: for TGM3, the forward primer was 5′-TCAACTGGCAGACGGCCTTCA-3′ and the reverse was 5′-GTACCGTCCTATGGGTGCGCT-3′; for β-actin, the forward primer was 5′-TCACCCACACTGTGCCCATCTACGA-3′, and the reverse primer was 5′-CAGCGGAACCGCTCATTGCCAATGG-3′. An initial denaturation step was performed for 5 minutes at 96°C, and 35 cycles were performed with the following PCR program: denaturing at 96°C for 15 seconds, annealing at 56°C for 30 seconds, and elongating at 72°C for 50 seconds. The program was completed with a final extension step at 72°C for 5 minutes.

### Real-time polymerase chain reaction analysis

All real-time PCR reactions were performed using an ABI 7300 real-time PCR system (Applied Biosystems, USA) and the SYBR Premix Ex Taq reagent kit (Takara). The primer sequences were as follows: for TGM3, the forward primer was 5′-GACAAGTTCTCCAGCCAGGAG-3′, and the reverse primer was 5′-AGTGGAAACACAGCCTTCGTC-3′; for β-actin, the forward primer was 5′-CCTGGCACCCAGCACAAT-3′, and the reverse primer was 5′-GGGCCGGACTCGTCATACT-3′. Real-time PCR was performed in a final volume of 20 μl with 1 μl of template cDNA at a concentration of 20 ng/μl with 10 μl SYBR Premix Ex Taq, 0.4 μl ROX Reference Dye and 0.2 μM of each primer for the target gene and the internal control (β-actin). Thermal cycling conditions were 95°C for 30 seconds followed by 40 cycles of 95°C for 5 seconds and 60°C for 31 seconds. The results of real-time PCR were defined as Ct values, which represent the cycle number at which the fluorescent signal of the samples passes a given threshold above the baseline. ΔCt was the difference in the Ct values derived from the specific genes compared with β-actin of the same sample. The relative expression level was determined as 2^-ΔΔCt^, where ΔΔCt = ΔCt (HNSCC samples)-ΔCt (adjacent normal sample), thereby indicating the fold change in the HNSCC samples relative to adjacent normal samples. The significance level was set at *P* < 0.05.

### Induction of gene expression by 5-Aza-dC and TSA

As previously described [46], to induce TGM3 expression, the above-mentioned HNSCC cell lines were treated with 2 μM or 5 μM 5′-aza-2′-deoxycytidine (5-Aza-dC), a DNA demethylation reagent, for 120 hours and 0.5 μM trichostatin A (TSA), a specific inhibitor of histone deacetylase for 48 hours.

### Cytosine methylation analysis

MethPrimer online software (http://www.urogene.org/index.html) was used to discover the CpG islands of the TGM3 promoter and to design bisulfate-sequencing PCR (BSP) primers. Genomic DNAs of HNSCC samples and cell lines were treated with the MethylCodeTM Bisulfite Conversion Kit (Invitrogen) and the converted DNA was subjected to PCR using BSP primers (forward, 5′-GTTTAGGTTGGAGTGTAGTGATG-3′; reverse, 5′-TCACTTAAAACCAAAAATTCAAAAC-3′). The PCR products were visualized by 2% agarose gel electrophoresis and subcloned into a pMD18-T vector (Takara). Ten colonies were sequenced using an ABI 3730 sequencer.

### Plasmid construction

To construct the TGM3 expression vector, the open reading frame (ORF) of human TGM3 cDNA was cloned into the eukaryotic expression vector pcDNA3.0 (Invitrogen). The forward primer used for the amplification of the ORF of the TGM3 cDNA was 5′-TTAAAGCTTCTGAGAAGAGGCAGAGGAAG-3, containing a HindIII site; the reverse primer was 5′-TATGAATTCTGTACGGGAGGCCACCAGCGC- 3, containing an EcoRI site without the stop codon. The amplified product of the TGM3 gene was purified, digested and ligated into the respective HindIII and EcoRI sites in the pcDNA3.0 vector.

### Adenoviral vectors

AdCMV-TGM3, a recombinant adenovirus expressing the TGM3, was constructed in a replication-deficient E1 and E3 adenovirus vector. Briefly, the complete coding sequence of the TGM3 gene was subcloned into the AdCMV vector. Then, the recombinant clones of shuttle plasmid and viral-backbone plasmid were packaged in HEK293 cells. The viral particles were purified by cesium chloride density gradient centrifugation and titered using the End-Point Dilution Assay. AdCMV-GFP, which only expresses GFP, was used as the control adenovirus.

### Western blot analysis

Cells were harvested in SDS lysis buffer (Beyotime, China), and cell lysates were electrophoresed through 10%-15% polyacrylamide gels and transferred to a nitrocellulose membrane. The membrane was incubated with a rabbit polyclonal TGM3 antibody (Sigma, USA 1:250), a rabbit polyclonal poly (ADP-ribose) polymerase (PARP) antibody, which recognize the 116-kDa full-length PARP protein (Abcam, USA 1:200), a rabbit monoclonal PARP antibody, which recognizes the 85-kDa cleaved PARP protein (Epitomics, USA 1:1000), a rabbit polyclonal caspase-8 antibody, which recognizes the 55-kDa procaspase-8 protein (Abcam, USA 1:1000), a rabbit polyclonal caspase-3 antibody, which recognizes the 32-kDa procaspase-3 protein (Abcam, USA 1:500), a rabbit monoclonal Bcl-2 antibody (Epitomics, USA 1:500) and a rabbit monoclonal Bax antibody (Epitomics, USA 1:500). A mouse monoclonal GAPDH antibody (Sigma, USA, 1:10,000) was used throughout as a loading control. The secondary antibodies were labeled with IRDye™800, and signals were observed using an Odyssey Infrared Imaging System (Rockland, USA).

### Cell proliferation assay

Cell-proliferation assays were performed to analyze the proliferation ability of WSU-HN4, HN13 and HN30 cells transiently transfected with the pcDNA3.0-TGM3 vector or the pcDNA3.0 empty vector. Briefly, the cells were plated in 96-well plates at 3 × 10^3^ cells per well and maintained at 37°C in a humidified incubator. Ten microliters of Cell-Counting Kit (CCK)-8 (Dojindo, Japan) solution was added at 24, 48, 72, 96 and 120 hours into triplicate wells and incubated for 1.5 hours. The number of viable cells in each well was measured by reading the optical density (OD) of absorbance at 450 nm. Measurements were performed in triplicate.

### Colony formation assay

Twenty-four hours after transiently transfecting with the pcDNA3.0-TGM3 vector or the pcDNA3.0 empty vector, 1 × 10^3^ cells were plated in 60-mm culture dishes and incubated with 500 μg/ml G418 for 20 days to allow for colony formation. The colonies were fixed with 70% ethanol for 1 hour, stained with 0.1% Coomassie Brilliant Blue R-250 (Thermo, USA) for 2 hours and washed with PBS. Colonies of more than 50 cells were counted under a dissecting microscope. The data from the colony formation assays were calculated as the means (±SD) from 3 independent experiments, each performed in triplicate.

### Apoptosis assay

The cells transiently transfected with the pcDNA3.0-TGM3 vector or pcDNA3.0 empty vector were harvested at 24, 48 and 72 hours. These cells were then quantified by flow cytometry using the FITC Annexin V Apoptosis Detection Kit (BD Biosciences, USA) according to the manufacturer’s protocols. Briefly, trypsinized adherent cells and floating cells were harvested, washed twice with cold PBS and resuspended in 1 × Binding Buffer at a concentration of 1 × 10^6^ cells/ml. Then, 5 μl of FITC Annexin V and 5 μl propidium iodide (PI) were added, and the cells were incubated for 15 minutes at 25°C in the dark. The cells were then resuspended in 400 μl of 1 × Binding Buffer and analyzed immediately by BD LSR II flow cytometry (BD Biosciences, USA).

### Tumorigenicity assay *in vivo*

To evaluate the anti-tumor effect of TGM3 *in vivo*, a HNSCC xenograft model was generated in BALB/C nude mice (4 weeks old) by subcutaneously injecting 4 × 10^6^ HN30 cells into the left and right axilla. One week after the injections, all mice were divided randomly into 2 groups (6 mice per group) and were treated with a multiple-center intratumor injection of 1 × 10^9^ Pfu (plaque forming units) of AdCMV-TGM3 or AdCMV-GFP 2 times a week for up to 2 weeks. One week after the treatments, the animals were sacrificed, and the tumor volume and tumor weight were measured. Differences in tumor weights were analyzed using Student’s *t*-test.

### Statistical analysis

For immunohistochemical analysis, the Student *t*-test and one-way ANOVA were used to analyze the associations between TGM3 expression levels and patient characteristics. The log-rank test was used to analyze univariate associations between the expression levels of TGM3 and overall survival. The Cox proportional hazards model was used for multivariate analyses, and all potential prognostic factors with *P* values < 0.05 from the univariate analysis were incorporated into multivariate analyses. The results of the real-time PCR, cell proliferation assay, colony formation assay and apoptosis assay were evaluated using Student’s *t*-tests. All tests were two-sided, and *P* values less than 0.05 were considered statistically significant.

## Abbreviations

TGM3: Transglutaminase 3; HNC: Head and neck cancer; HNSCC: Head and neck squamous cell carcinoma; OSCC: Oral squamous cell carcinoma; ESCC: Esophageal squamous cell carcinoma; TSS: Transcriptional start site; TGMs: Transglutaminases; CI: Confidence interval; HR: Hazard ratio; pN: Pathological lymph node status; TNM: Tumor-lymph node-metastasis classification; ATCC: American Type Culture Collection; ABC: Avidin-biotin complex; IOD: Integrated optical density; 5-Aza-dC: 5′-aza-2′-deoxycytidine; TSA: Trichostatin A; BSP: Bisulfate-sequencing PCR; ORF: Open reading frame; PI: Propidium iodide.

## Competing interests

The authors declare that they have no competing interests.

## Authors’ contributions

XBW, WC, XW, JJZ, QX, ZJL and WTC performed experiments; WTC, YDW and JJZ participated in the design of the study; XBW, XW and WC participated in data analysis and statistical analysis; XBW, YDW and WTC drafted and revised the manuscript. All authors read and approved the final manuscript.

## Supplementary Material

Additional file 1: Table S1 Clinicopathologic characteristics of 101 patients. Click here for file
