# Supplementary material for: TGM3, a candidate tumor suppressor gene, contributes to human head and neck cancer
Source: Mol Cancer. 2013 Dec 1;12:151. doi: 10.1186/1476-4598-12-151 (PMC4176127; doi:10.1186/1476-4598-12-151)
Supplement: Additional file 1: Table S1 — Clinicopathologic characteristics of 101 patients. [file 1476-4598-12-151-S1.doc]

Table S1 Clinicopathologic Characteristics of 101 Patients

| Characteristic | Classification | Case number | Percentage(%) |
| --- | --- | --- | --- |
| Age, years | Range | 28-75 |  |
|  | Mean±SD | 54.3±11.8 |  |
|  | Median | 54 |  |
|  | <60 | 37 | 36.6 |
|  | ≥60 | 64 | 63.4 |
| Sex | Men | 53 | 52.5 |
|  | Women | 48 | 47.5 |
| Alcohol history | Drinker | 22 | 21.8 |
|  | Nondrinker | 79 | 78.2 |
| Smoking history | Smoker | 32 | 31.7 |
|  | Nonsmoker | 69 | 68.3 |
| Tumor grade | Well differentiated | 64 | 63.4 |
|  | Moderately differentiated | 26 | 25.7 |
|  | Poorly differentiated | 11 | 10.9 |
| TNM stage | I | 16 | 15.8 |
|  | II | 35 | 34.7 |
|  | III | 26 | 25.7 |
|  | IV | 24 | 23.8 |
| Lymph node metastasis | pN0 | 69 | 68.3 |
|  | pN1-pN2 | 32 | 31.7 |
| Disease site | Tongue | 76 | 75.2 |
|  | Cheek | 12 | 11.9 |
|  | Gingive | 10 | 9.9 |
|  | Orther | 3 | 3 |
